# Dataset on the effects of spermidine on linking number differences between histone H1-free and histone H1-bound circular polynucleosomes

**DOI:** 10.1016/j.dib.2018.01.091

**Published:** 2018-02-03

**Authors:** Hao Zhang, Tianhu Li

**Affiliations:** Division of Chemistry and Biological Chemistry, School of Physical and Mathematical Sciences, Nanyang Technological University, 21 Nanyang Link, Singapore 637371, Singapore

## Abstract

The data presented in this article are related to the research article entitled "Quantitative determination of linking number differences between circular polynucleosomes and histone H1-bound circular polynucleosomes" Zhang et al. (in press) [1]. DNA linking number differences between histone H1-free and histone H1-bound circular polynucleosomes at various spermidine concentrations was quantitatively determined by chloroquine-based gel electrophoretic analysis in this work, which provides information on the topological effects of histone H1 and spermidine on the linker DNA between nucleosomes.

**Specifications Table**

**Table:** 

Subject area	*Biochemistry*
More specific subject area	*DNA linking number, Nucleosome, Histone H1*
Type of data	*Graph, figure*
How data was acquired	*Electrophoretic data were acquired by G:BOX iChemi gel documentation system (Syngene), quantified by GeneTools 4.01 (Syngene), and analyzed by OriginPro 9.1 (OriginLab Corporation).*
Data format	*Raw, analyzed*
Experimental factors	*The histones, enzymes and plasmid DNA were purchased from New England Biolabs Inc. Polynucleosomes were prepared following the Dilution Assembly Protocol (E5350) provided by New England Biolabs Inc.*
Experimental features	*DNA linking number distribution was determined by agarose electrophoresis at 1.5 V/cm in 1× TAE buffer for 12 h in the presence of 1.2 μg/ml of chloroquine.*
Data source location	*Nanyang Technological University, Singapore*
Data accessibility	*Data within this and the main article.*
Related research article	*H. Zhang, T. Li, Quantitative determination of linking number differences between circular polynucleosomes and histone H1-bound circular polynucleosomes, Bioorg. Med. Chem. Lett. (2017) (in press).*

**Value of the data**

• Contribution of histone H1 to DNA linking number is quantitatively measured, which could be valuable for elucidation of the roles of linker histones in spatial arrangement of chromatin.
• Concentration-dependent effects of spermidine on DNA linking number of polynucleosomes is of value for the experimental design in the future biochemical and biomedical research.
• The chloroquine-based electrophoresis method could be useful for determination of DNA linking number differences and analysis of DNA topoisomer distributions.

## 1 Data

**Fig. 1:**
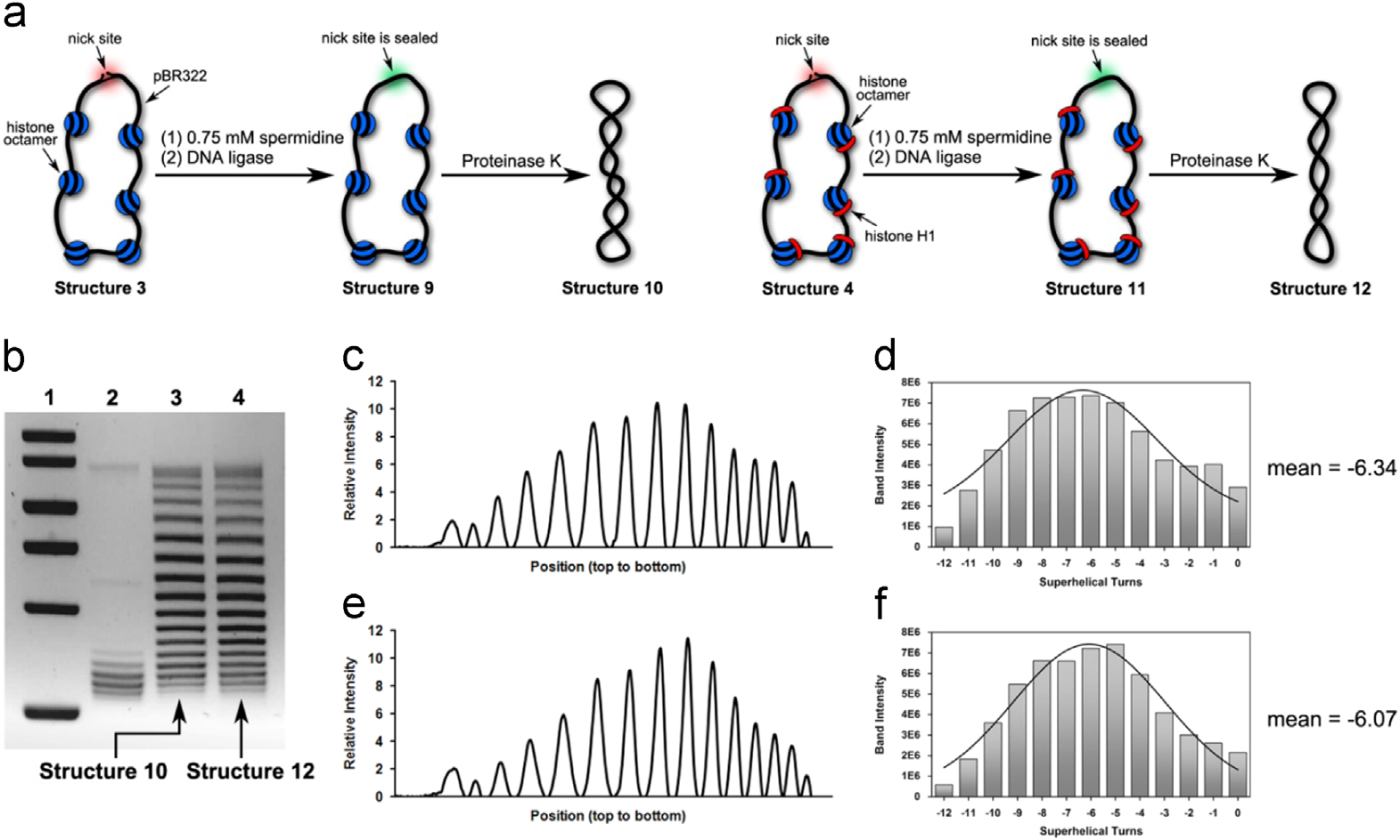
(a) Illustration of our preparations of Structure 10 and Structure 12 in the presence of 0.75 mM spermidine. (b) Chloroquine-based agarose gel electrophoretic analysis on Structure 10 and Structure 12. Lane 1: molecular weight markers; Lane 2: relaxed forms of pBR322; Lane 3: Structure 10 and Lane 4: Structure 12. (c) Densitometry tracing of gel electrophoretic results in Lane 3. (d) Plot of Gauss fit on the data shown in Fig. 1c, which gave rise to − 6.34 as its mean value of *ΔLk* (e) Densitometry tracing of gel electrophoretic results in Lane 4. (f) Plot of Gauss fit on the data shown in Fig. 1e, which gave rise to − 6.07 as its mean value of *ΔLk*.

**Fig. 2:**
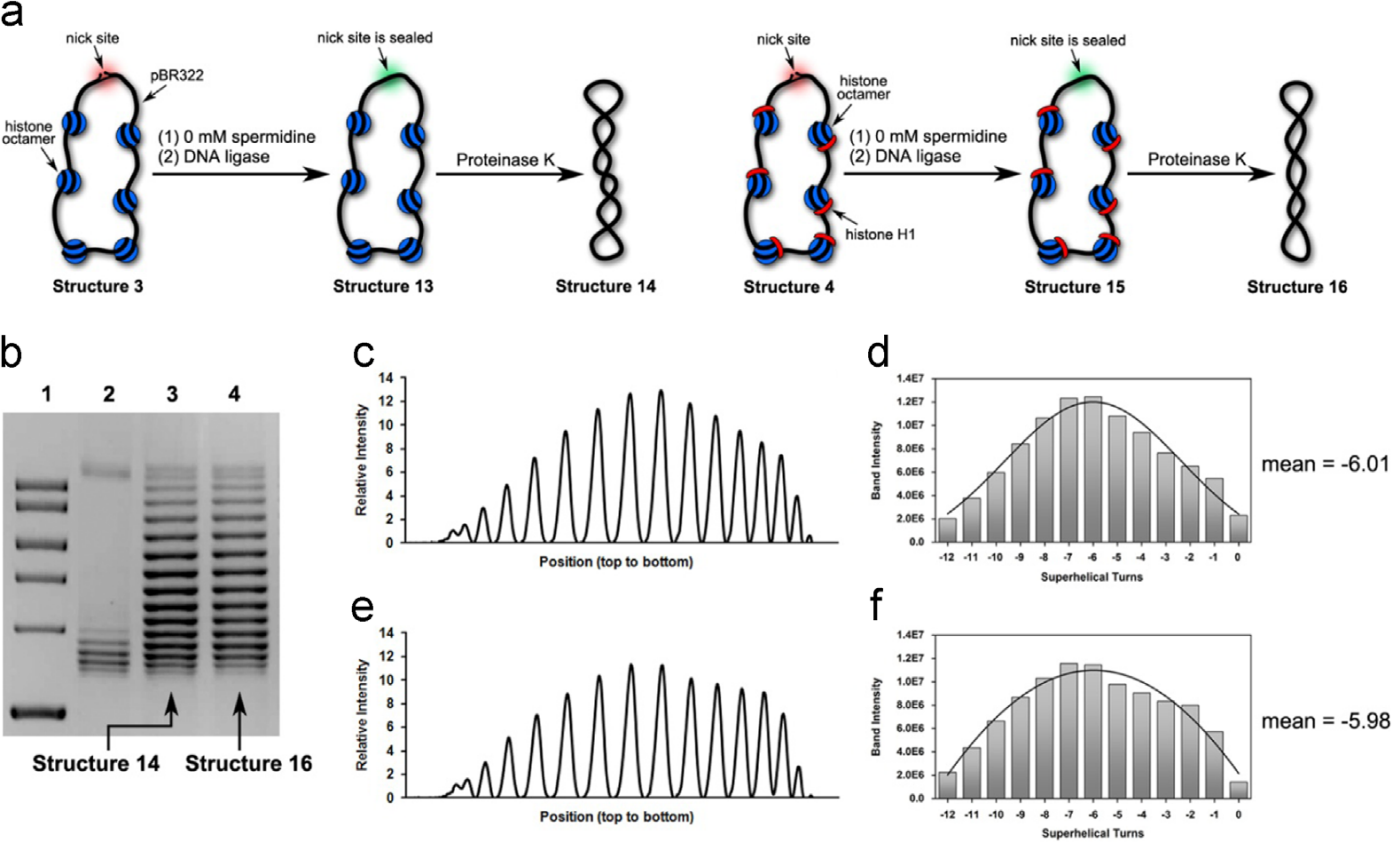
(a) Illustration of our preparations of Structure 14 and Structure 16 in the presence of 0 mM spermidine. (b) Chloroquine-based agarose gel electrophoretic analysis on Structure 14 and Structure 16. Lane 1: molecular weight markers; Lane 2: relaxed forms of pBR322; Lane 3: Structure 14 and Lane 4: Structure 16. (c) Densitometry tracing of gel electrophoretic results in Lane 3. (d) Plot of Gauss fit on the data shown in Fig. 2c, which gave rise to − 6.01 as its mean value of *ΔLk* (e) Densitometry tracing of gel electrophoretic results in Lane 4. (f) Plot of Gauss fit on the data shown in Fig. 2e, which gave rise to − 5.98 as its mean value of *ΔLk*.

**Fig. 3:**
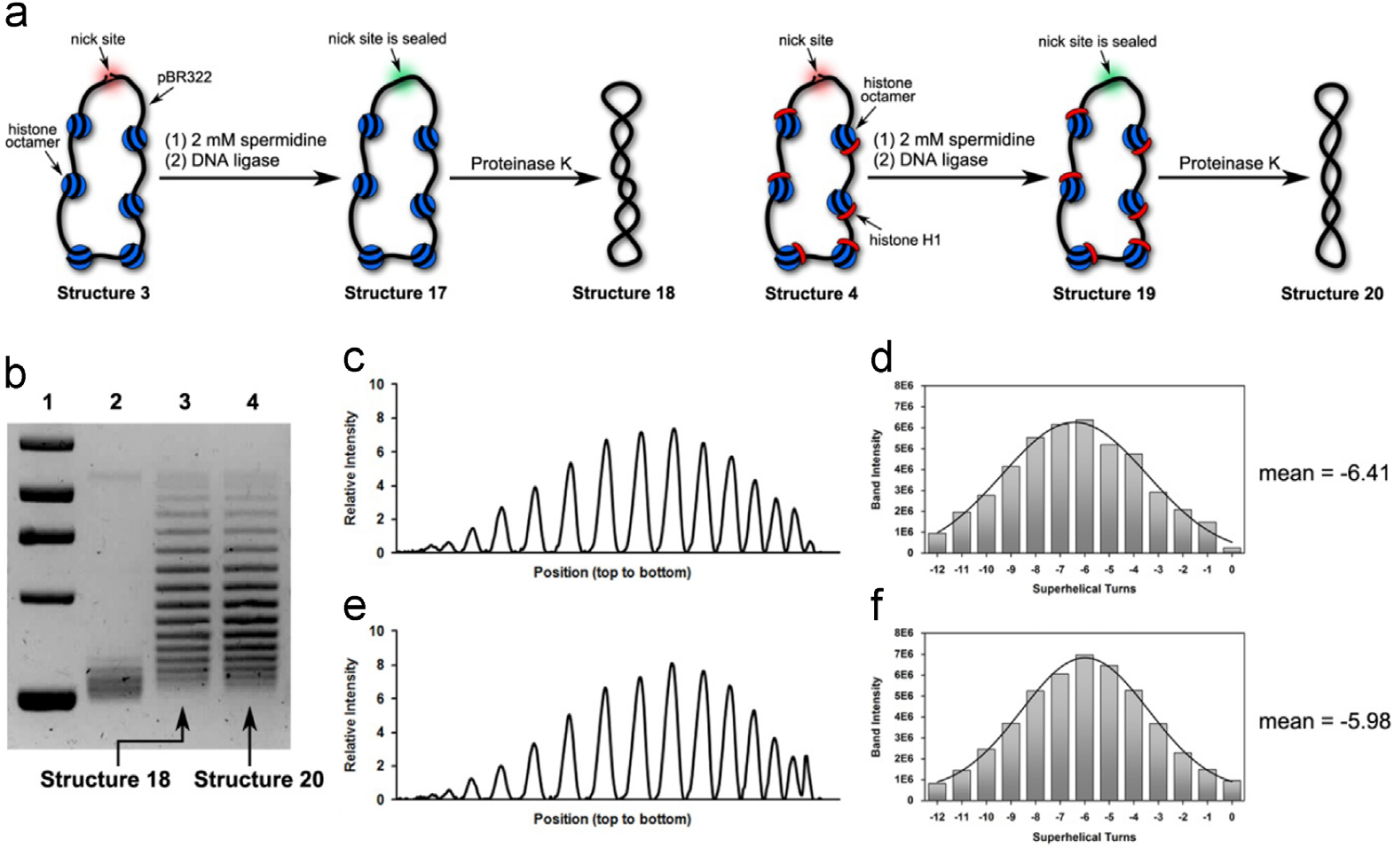
(a) Illustration of our preparations of Structure 18 and Structure 20 in the presence of 2 mM spermidine. (b) Chloroquine-based agarose gel electrophoretic analysis on Structure 18 and Structure 20. Lane 1: molecular weight markers; Lane 2: relaxed forms of pBR322; Lane 3: Structure 18 and Lane 4: Structure 20. (c) Densitometry tracing of gel electrophoretic results in Lane 3. (d) Plot of Gauss fit on the data shown in Fig. 3c, which gave rise to − 6.41 as its mean value of *ΔLk* (e) Densitometry tracing of gel electrophoretic results in Lane 4. (f) Plot of Gauss fit on the data shown in Fig. 3e, which gave rise to − 5.98 as its mean value of *ΔLk*.

**Fig. 4:**
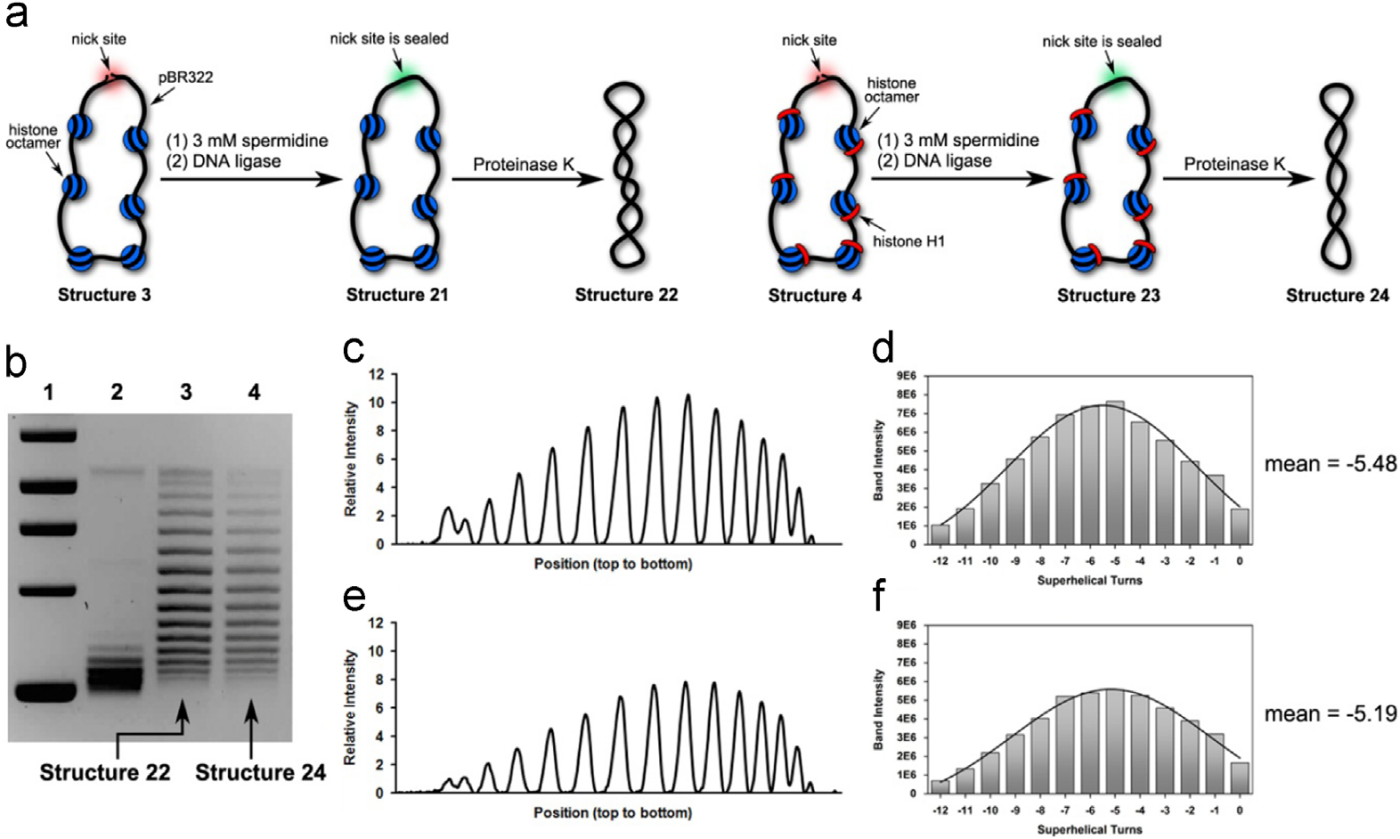
(a) Illustration of our preparations of Structure 22 and Structure 24 in the presence of 3 mM spermidine. (b) Chloroquine-based agarose gel electrophoretic analysis on Structure 22 and Structure 24. Lane 1: molecular weight markers; Lane 2: relaxed forms of pBR322; Lane 3: Structure 22 and Lane 4: Structure 24. (c) Densitometry tracing of gel electrophoretic results in Lane 3. (d) Plot of Gauss fit on the data shown in Fig. 4c, which gave rise to − 5.48 as its mean value of *ΔLk* (e) Densitometry tracing of gel electrophoretic results in Lane 4. (f) Plot of Gauss fit on the data shown in Fig. 4e, which gave rise to − 5.19 as its mean value of *ΔLk*.

**Fig. 5:**
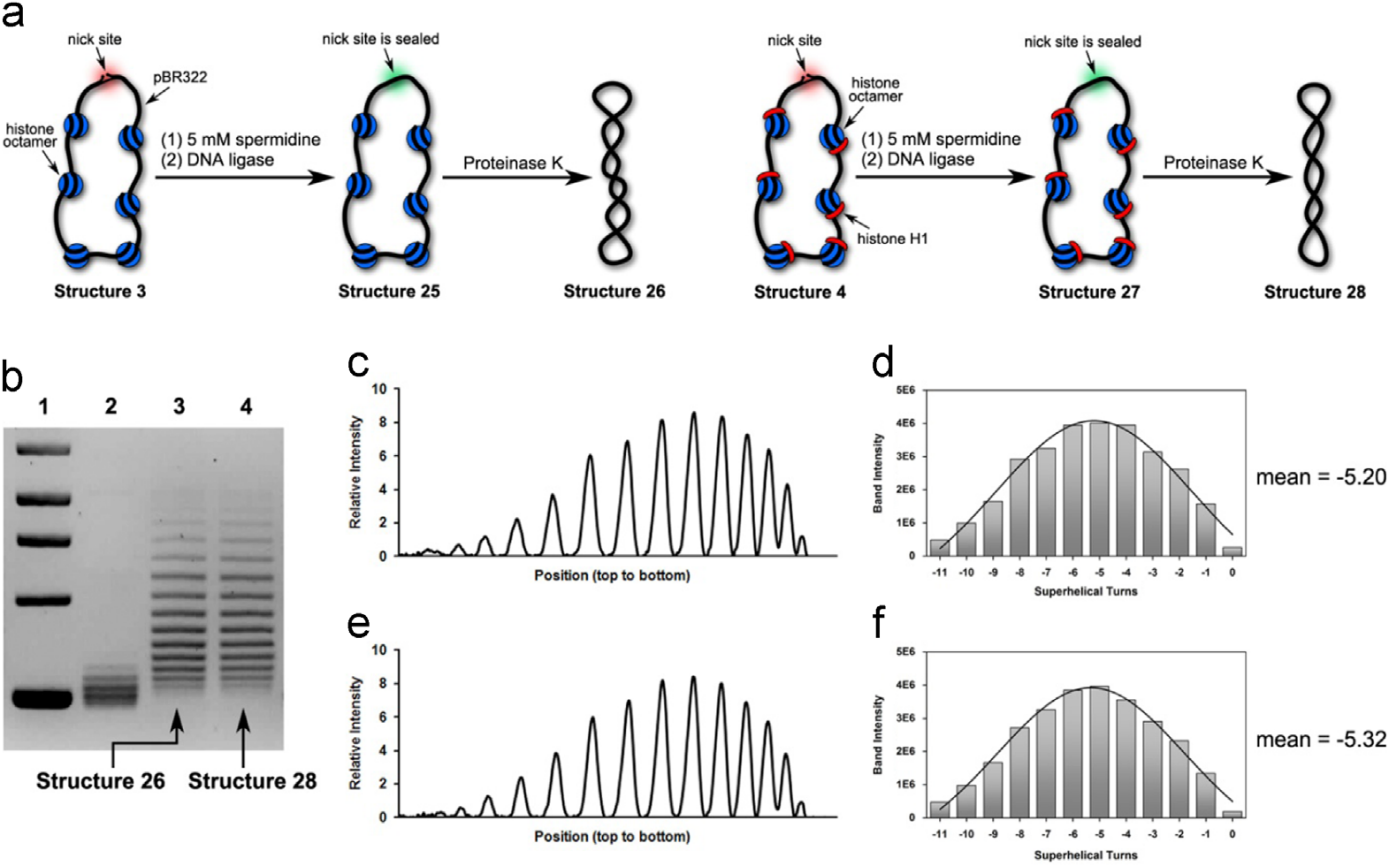
(a) Illustration of our preparations of Structure 26 and Structure 28 in the presence of 5 mM spermidine. (b) Chloroquine-based agarose gel electrophoretic analysis on Structure 26 and Structure 28. Lane 1: molecular weight markers; Lane 2: relaxed forms of pBR322; Lane 3: Structure 26 and Lane 4: Structure 28. (c) Densitometry tracing of gel electrophoretic results in Lane 3. (d) Plot of Gauss fit on the data shown in Fig. 5c, which gave rise to − 5.20 as its mean value of *ΔLk* (e) Densitometry tracing of gel electrophoretic results in Lane 4. (f) Plot of Gauss fit on the data shown in Fig. 5e, which gave rise to − 5.32 as its mean value of *ΔLk*.

**Fig. 6:**
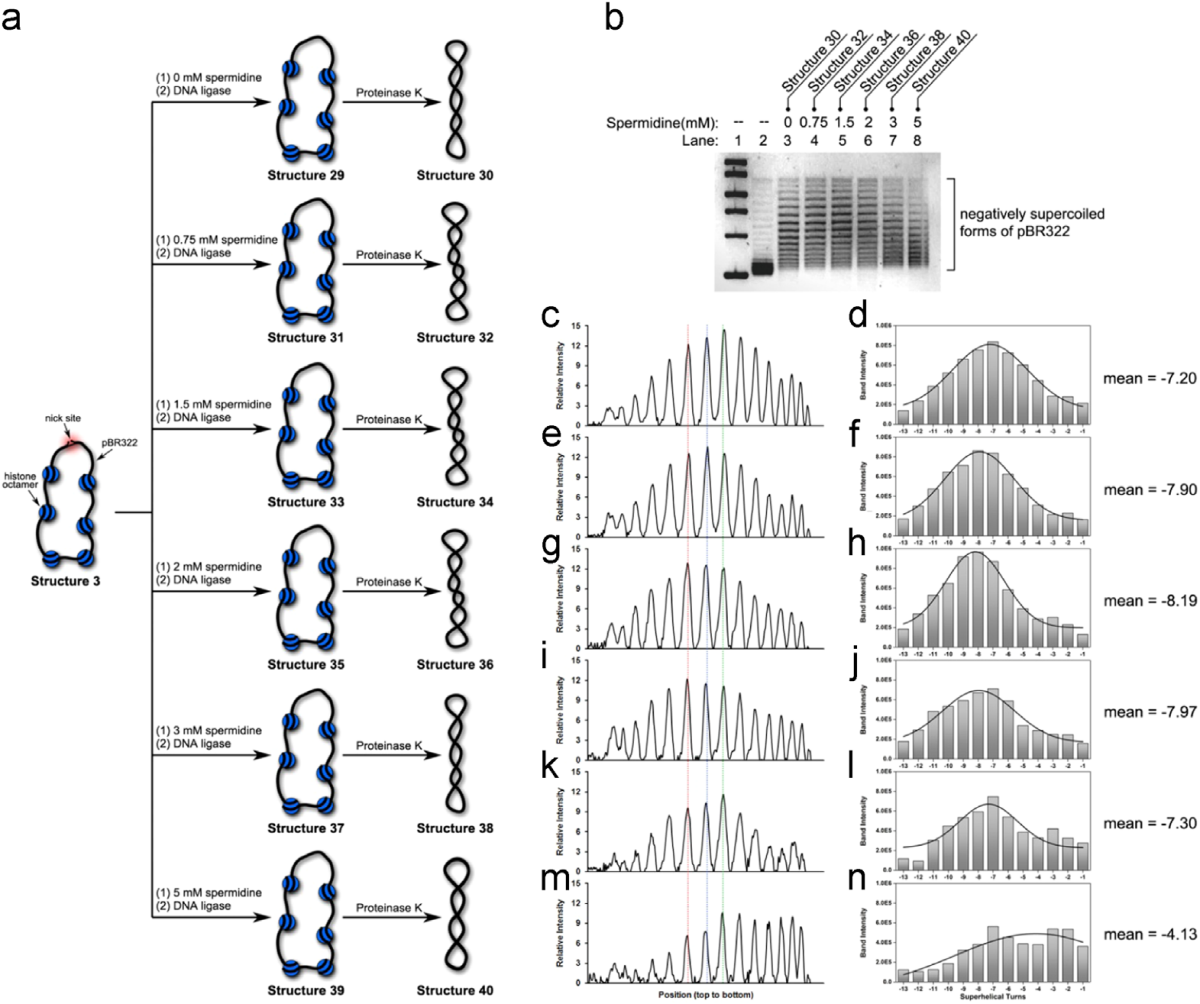
(a) Illustration of our preparations of Structure 30, Structure 32, Structure 34, Structure 36, Structure 38 and Structure 40 in the presence of increasing amount of spermidine. (b) This figure is same as Fig. 6(a) in Ref. [1]. (c) Densitometry tracing of Lane 3. (d) Plot of Gauss fit on the data shown in (c), which gave rise to − 7.20 as its mean value of *ΔLk*. (e) Densitometry tracing of Lane 4. (f) Plot of Gauss fit on the data shown in (e), which gave rise to − 7.90 as its mean value of *ΔLk*. (g) Densitometry tracing of Lane 5. (h) Plot of Gauss fit on the data shown in (g), which gave rise to − 8.19 as its mean value of *ΔLk*. (i) Densitometry tracing of Lane 6. (j) Plot of Gauss fit on the data shown in (i), which gave rise to − 7.97 as its mean value of *ΔLk*. (k) Densitometry tracing of Lane 7. (l) Plot of Gauss fit on the data shown in (k), which gave rise to − 7.30 as its mean value of *ΔLk*. (m) Densitometry tracing of Lane 8. (n) Plot of Gauss fit on the data shown in (m), which gave rise to − 4.13 as its mean value of *ΔLk*.

It has been reported in Ref. [1] that binding of histone H1 molecules produces linking number changes in circular polynucleosomes in the presence of 1.5 mM spermidine [1]. With the aim of knowing the effects of spermidine concentration on the linking number changes caused by histone H1, various amounts of spermidine (0 mM, 0.75 mM, 2 mM, 3 mM, 5 mM) were incubated with the open-circular polynucleosomes respectively before ligation reactions. The distribution patterns of these obtained DNA topoisomers were subsequently resolved by chloroquine-based gel electrophoresis (data presented in Fig. 1, Fig. 2, Fig. 3, Fig. 4, Fig. 5). In addition, effects of spermidine alone (without involvement of histone H1) on the linking number of circular polynucleosomes were determined and presented in Fig. 6 in this article as well.

## 2 Experimental design, materials, and methods

### 2.1 Construction of open-circular polynucleosomes

Preparation of open-circular histone H1-free polynucleosome (Structure 3) and open-circular histone H1-bound polynucleosome (Structure 4) was carried out though the following procedures which has been described in Ref. [1] as well:

**Step 1:** One of the DNA strands was cleaved at a specific recognition sequence by the action of DNA nicking endonuclease. 5 μg pBR322 (Structure 1) was cleaved by 5 U of Nt.BspQI nicking endonuclease at 50 °C over 2 hours in a 50 μl buffer solution (50 mM Tris–HCl pH 7.9, 100 mM NaCl, 10 mM MgCl_2_, 1 mM DTT). The obtained reaction products (Structure 2) were subsequently purified by QIAquick PCR Purification Kit.
**Step 2:** Open-circular polynucleosomes (Structure 3) were constructed following the manufacturer's protocol and our previous experimental procedures [2], [3]. The open-circular plasmid DNA was incubated with histone H2A/H2B dimer and histone H3.1/H4 tetramer in the presence of 2 M NaCl. This 10 μl mixture was then stepwisely diluted with 10 mM Tris–HCl buffer (pH 8.0) to final volume of 400 μl.
**Step 3:** Histone H1-bound polynucleosomes (Structure 4) were prepared as described in our previous work [2], [3]. The open-circular polynucleosomes was incubated with 1 μg/ml of recombinant human histone H1° at room temperature for 30 minutes.

### 2.2 Construction of close circular polynucleosomes

Open-circular polynucleosomes (Structure 3 or Structure 4) were incubated with certain amount of spermidine at room temperature for 30 min. 100 units of T4 DNA ligase were then incubated with these open-circular polynucleosomes at room temperature for 2 hours in the presence of 50 mM Tris–HCl (pH 7.5), 10 mM MgCl_2_, 1 mM ATP and 10 mM DTT. The reaction products were treated with 10 units of Proteinase K for 30 min. After removal of proteins by QIAquick PCR Purification Kit, the circular DNA were then incubated with 30 units of *Escherichia coli* Exonuclease III in the presence of 10 mM Bis-Tris-Propane-HCl (pH 7.0), 10 mM MgCl2 and 1 mM DTT at 37 °C for 30 min before gel electrophoretic analysis.

### 2.3 Determination of DNA linking number changes by gel electrophoresis

Electrophoretic examinations were carried out on 1.4% agarose gel (with 1.2 μg/ml chloroquine inside the gel) at 1.5 V/cm. Gel was stained with ethidium bromide and subsequently visualized using G:BOX iChemi gel documentation system. Distribution of DNA bands was quantified using GeneTools 4.01. Gauss fit was performed by OriginPro 9.1 using the same approach as previously reported [4], [5], [6], [7].

### 2.4 Materials

Recombinant human histone H2A/H2B dimer, histone H3.1/H4 tetramer, histone H1^0^, pBR322 vector, Nt.BspQI nicking endonuclease, T4 DNA ligase and Proteinase K were purchased from New England Biolabs Inc. (Singapore). QIAquick PCR Purification Kit was obtained from QIAGEN Singapore Pte. Ltd. (Singapore). Tris–HCl buffer, Tris-acetate-EDTA (TAE) buffer and agarose were provided by Axil Scientific Pte. Ltd. (Singapore). Ethidium bromide was purchased from Bio-Rad Laboratories Inc. (Singapore). Spermidine was obtained from Sigma-Aldrich Pte. Ltd. (Singapore). G:BOX iChemi gel documentation system was purchased from Syngene Inc. (Cambridge, UK). Other salts and chemicals used in this research were obtained from Sigma-Aldrich Pte. Ltd. (Singapore) with analytical grade or molecular biology grade.

## References

[1] Zhang H., Li T. (2018). Quantitative determination of linking number differences between circular polynucleosomes and histone H1-bound circular polynucleosomes. Bioorg. Med. Chem. Lett..

[2] Zhang H., Li T. (2017). Presence of negative supercoiling in aggregates of histone H1-plasmidic polynucleosome complexes. Bioorg. Med. Chem. Lett..

[3] Zhang H., Li T. (2017). Effects of spermidine and ATP on stabilities of chromatosomes and histone H1-depleted chromatosomes. Bioorg. Med. Chem. Lett..

[4] Vetcher A.A., McEwen A.E., Abujarour R., Hanke A., Levene S.D. (2010). Gel mobilities of linking-number topoisomers and their dependence on DNA helical repeat and elasticity. Biophys. Chem..

[5] Bates Andrew D., Maxwell A. (2010). The role of ATP in the reactions of type II DNA topoisomerases. Biochem. Soc. Trans..

[6] Son L.S., Bacolla A., Wells R.D. (2006). Sticky DNA: in vivo formation in E. coli and in vitro association of long GAA*TTC tracts to generate two independent supercoiled domains. J. Mol. Biol..

[7] Hardin A.H., Sarkar S.K., Seol Y., Liou G.F., Osheroff N., Neuman K.C. (2011). Direct measurement of DNA bending by type IIA topoisomerases: implications for non-equilibrium topology simplification. Nucleic Acids Res..

